# Nuclear–mitochondrial epistasis: a gene's eye view of genomic conflict

**DOI:** 10.1002/ece3.2345

**Published:** 2016-08-18

**Authors:** Michael J. Wade, Devin M. Drown

**Affiliations:** ^1^ Department of BiologyIndiana UniversityBloomingtonIndiana47405; ^2^ Institute of Arctic BiologyUniversity of Alaska FairbanksFairbanksAlaska99775

**Keywords:** Adaptation, coevolution, cooperation, population genetics

## Abstract

We use population genetic models to investigate the cooperative and conflicting synergistic fitness effects between genes from the nucleus and the mitochondrion. By varying fitness parameters, we examine the scope for conflict relative to cooperation among genomes and the utility of the “gene's eye view” analytical approach, which is based on the marginal average fitness of specific alleles. Because sexual conflict can maintain polymorphism of mitochondrial haplotypes, we can explore two types of evolutionary conflict (genomic and sexual) with one epistatic model. We find that the nuclear genetic architecture (autosomal, X‐linked, or Z‐linked) and the mating system change the regions of parameter space corresponding to the evolution by sexual and genomic conflict. For all models, regardless of conflict or cooperation, we find that population mean fitness increases monotonically as evolution proceeds. Moreover, we find that the process of gene frequency change with positive, synergistic fitnesses is self‐accelerating, as the success of an allele in one genome or in one sex increases the frequency of the interacting allele upon which its success depends. This results in runaway evolutionary dynamics caused by the positive intergenomic associations generated by selection. An inbreeding mating system tends to further accelerate these runaway dynamics because it maintains favorable host–symbiont or male–female gene combinations. In contrast, where conflict predominates, the success of an allele in one genome or in one sex diminishes the frequency of the corresponding allele in the other, resulting in considerably slower evolutionary dynamics. The rate of change of mean fitness is also much faster with positive, synergistic fitnesses and much slower where conflict is predominant. Consequently, selection rapidly fixes cooperative gene combinations, while leaving behind a slowing evolving residue of conflicting gene combinations at mutation–selection balance. We discuss how an emphasis on marginal fitness averages may obscure the interdependence of allelic fitness across genomes, making the evolutionary trajectories appear independent of one another when they are not.

## Introduction

Functional interactions between proteins encoded by the nuclear and mitochondrial genomes, such as those of the human cytochrome *c* oxidase complex (Poyton 1998), require the coordinated expression of tens of nuclear and several organelle genes. This interaction has been called “the most significant coevolved mutualism in the history of life” (Rand et al. 2001). One evolutionary explanation for this intimate coadaptation between genomes is cooperative evolution, wherein favorable nuclear–mitochondrial gene combinations experienced positive selection and deleterious intergenomic combinations were selected against (Wade and Goodnight 2006; Brandvain and Wade 2009). The existence of extensive intergenomic functional coordination is taken as an evidence for cooperative evolution (Blier et al. 2001). Additional supporting evidence comes from hybridization which tends to disrupt coadaptation by bringing together nuclear–mitochondrial gene combinations untested by natural selection and whose dysfunction lowers the viability or fertility fitness of hybrids.

An alternative explanation emerges from the “gene's eye view” of evolution (Dawkins 1976). In this view, the nuclear and mitochondrial genomes have been involved in an evolutionary arms race, wherein the independent evolution of genes in one genome has favored maximization of its fitness, sometimes at the expense of genes in the other genome. As a result, each genome evolved reciprocally to limit the negative fitness effects on itself of adaptation by the other. In this view, mitochondrion is an “encapsulated slave” (Maynard Smith and Szathmáry 1995, p. 141). With separate sexes, the intergenomic contest becomes a host sexual conflict because “…mitochondrial genes are selected only to increase female fitness, whereas nuclear genes are selected to increase male and female fitnesses equally. Thus, different patterns of transmission (uni‐ versus biparental) lead to conflicts over investment in male versus female function” (Burt and Trivers 2006, p. 143). Reciprocal coadaptation occurs between the two genomes or between the two sexes owing to conflicting rather than to cooperative fitness effects. With dual inheritance, where two species or genomes interact, evolutionary inference is based on the marginal fitnesses of single genes in each genome or in each sex. As in the cooperative paradigm, hybrid dysfunction, manifest more often by male hybrids than by females, is interpreted as an evidence of sexual and genomic conflict (Cosmides and Tooby 1981; Burt and Trivers 2006; Rice 2013). This has been stated as the principle that “nothing in genetics makes sense except in light of genomic conflict” (Rice 2013).

In this article, we examine the full spectrum of fitness interactions between genes from the nucleus and the mitochondrion, using population genetic models with both cooperative and conflicting synergistic fitness effects. Specifically, by varying fitness effects, we examine the relative scope for conflict relative to the cooperation among genes. And, by employing epistatic fitness effects, we examine the utility of the “gene's eye view” analytical approach, which is based on the marginal average fitness of specific alleles. Unlike epistasis between alleles at nuclear loci, mitochondrial–nuclear interactions can maintain polymorphism of mitochondrial haplotypes under only two conditions: (1) negative frequency‐dependent selection (Gregorius and Ross 1984) or (2) sexually antagonistic selection (Rand 2001). It is the latter which permits us to investigate the gene's eye view of sexual conflict by postulating nuclear alleles with sexually dimorphic effects on fitness depending on cytoplasmic background.

Although intergenomic adaptation can occur by either conflict or cooperation, we find that the region of parameter space corresponding to evolution by genomic cooperation is larger than that of genomic conflict. Moreover, the evolutionary dynamics of gene frequency change vary considerably in different sectors of the adaptive landscape. In regions with intergenomic fitness conflict, the rate of adaptive change is slow, because successful genes in one genome tend to lower the frequency of the genetic backgrounds in the other genome on which their evolutionary success depends and vice versa. In contrast, with positive synergistic fitnesses, adaptive change is self‐accelerating with runaway dynamics reminiscent of Fisherian sexual selection via female mate choice (Fisher 1958; Lande 1981; Kirkpatrick 1982; Breden and Wade 1991; Drown and Wade 2014). Because of the fitness synergy, selection creates genome associations, so that successful genes in one genome increase the frequency of the genetic backgrounds in the other genome on which their success depends and vice versa, resulting in a runaway evolution. We discuss how an emphasis on marginal fitness averages may obscure the interdependence of allelic fitness across genomes, making the evolutionary trajectories appear independent of one another when they are not.

## The Model

We model the case of two loci, one nuclear and one mitochondrial, and follow pioneering studies of nuclear‐cytoplasmic epistasis (Clark 1984; Asmussen et al. 1987; Arnold et al. 1988) by including positive and negative epistasis for fitness. By calculating the marginal fitnesses of the nuclear alleles and the mitochondrial genes, respectively, our model affords the gene's eye views of genomic conflict as well as sexual conflict. For the nuclear genome, we assume a diploid species with discrete nonoverlapping generations, where females mate multiply and at random. Later, we relax the random mating assumption to allow inbreeding by brother–sister mating to see whether or not it accelerates coadaptation between genomes in a manner similar to that between genes in the same genome (Breden and Wade 1991; Drown and Wade 2014).

We first consider diploids with alternative autosomal nuclear alleles, *A* and *a*, whose mitochondrial genotype is the alternative alleles, *C* and *c*. The frequency of the *A* allele in the population is *P*_*A*_ and that of the *C* allele is *P*_*C*_. The nuclear genotypes differ in fitness depending upon the mitochondrial genetic background at the *C* locus. The mean fitness for all individuals with the *C* mitochondrial background is changed by a selection coefficient, *β*, which can be negative, positive, or zero. We assume that alleles at the *A* locus act additively on this background so that *w*_*AAC*_, the fitness of an *AAC* genotypes, equals (1 + *β *+ 2*α*); *w*_*AaC*_ is (1 + *β *+ *α*); and *w*_*aaC*_ is (1 + *β*). In contrast, on the *c* mitochondrial background, all nuclear genotypes (*AAc*,* Aac*, and *aac*) have a fitness of 1. We have summarized these fitnesses in Table 1.

**Table 1:** Mitonuclear combinations and associated fitness

	Nuclear genotype		
	AA	Aa	aa
Mitochondrial genotype			
C	1 + *β *+ 2*α	1 + *β *+ *α*	1 + *β*
c	1	1	1

We allow males and females of the same genotype to have different fitnesses by subscripting the selection coefficient, *α*, as *α*_m_ and *α*_f_, for males and females, respectively. Thus, the marginal fitness of the *C* cytotype is beneficial to females, but detrimental to males whenever *α*_m_ < 0 < *α*_f_. This constitutes the classic case of intergenomic, cytonuclear conflict (Cosmides and Tooby 1981; Frank and Hurst 1996; Burt and Trivers 2006), known as mother's curse (Gemmell et al. 2004), wherein the matrilineal transmission of the mitochondria creates an “asymmetric sieve” favoring mitochondrial alleles advantageous for females (0 < *α*_f_), but harmful for males (*α*_m_ < 0), a finding. Moreover, we have the conditions for intralocus sexual conflict whenever *α*_f_ < 0 < *α*_m_ or *α*_m_ < 0 < *α*_f_. Thus, this simple epistatic model permits two gene's eye views, one for the marginal fitnesses of the nuclear alleles and one for the mitochondrial alleles. And, the condition, *α*_m_ < 0 < *α*_f_, results in both genomic and sexual conflicts.

We modeled other nuclear genetic architectures by allowing the *A* locus to be X‐linked or Z‐linked instead of autosomal as assumed above. For the X‐linked case, we assumed alternative nuclear alleles, *X* and *x*, with diploid females and haploid, heterogametic males. The fitnesses of female genotypes were identical to those of the autosomal case aforesaid. Male fitnesses were *w*_*XYC*_, equal to (1 + *β *+ *α*_*m*_), and *w*_*xYC*_ equal to (1 + *β*). (We assumed that the *XYC* male had 1/2 the expression level of an *XXC*, homozygous female.) For the Z‐linked case, we assumed alternative nuclear alleles, Z and z, with diploid males and haploid, heterogametic females. The female genotypic fitnesses were similar to the corresponding male genotypic fitnesses in the X‐linked case; that is, *w*_*ZWC*_, the fitness of a *ZWC* female genotype equaled (1 + *β *+ *α*_f_), and *w*_*zWC*_ was (1 + *β*). Male genotypic fitnesses in this Z‐linked case were identical to those of the autosomal case.

## Results

### One‐generation changes

To obtain analytic solutions, we assumed that the genotypes were the products of random mating and initially in multilocus Hardy–Weinberg proportions as is standard in population genetics theory (Charlesworth and Charlesworth 2010). In the following section, we relax these simplifying assumptions and explore numerical iterations of the exact equations with the addition of inbreeding by brother–sister mating. We also assumed that females mated multiply, so that all families consisted of maternal half‐sibs. With these assumptions, the exact change in the frequency of the *A* nuclear allele in each sex equals

(1a) $$\Delta P_{A,\mathrm{female}}=\frac{\alpha_{f}P_{C}}{[1+P_{C}\left(\beta +2\alpha_{f}P_{A}\right)]}P_{A}\left(1-P_{A}\right),$$

(1b) $$\Delta P_{A,\mathrm{male}}=\frac{\alpha_{m}P_{C}}{\left[1+P_{C}\left(\beta +2\alpha_{m}P_{A}\right)\right]}P_{A}\left(1-P_{A}\right).,$$

where the denominators are mean fitness in females, *w*_female_, and in males, *w*_male_, respectively. Sexual conflict occurs whenever *α*_f_ and *α*_m_ differ in sign (i.e., when *α*_f_ < 0 < *α*_m_ or when *α*_m_ < 0 < *α*_f_).

The change in the frequency of the *C* mitochondrial allele, *P*_*C*_, in each sex equals

(2a) $$\Delta P_{C,\mathrm{female}}=\frac{\left(\beta +2\alpha_{f}P_{A}\right)}{\left[1+P_{C}\left(\beta +2\alpha_{f}P_{A}\right)\right]}P_{C}\left(1-P_{C}\right),$$

(2b) $$\Delta P_{C,\mathrm{male}}=\frac{\left(\beta +2\alpha_{m}P_{A}\right)}{\left[1+P_{C}\left(\beta +2\alpha_{m}P_{A}\right)\right]}P_{C}\left(1-P_{C}\right).$$

Owing to maternal inheritance, the total change in *P*_*C*_ equals ∆*P*_*C,*female_, because males do not contribute mitochondria to the next generation. (Our model could accommodate “mitochondrial leakage” (Wade and McCauley 2005) from males to their offspring at a rate, *L*, by weighting the male and female contributions to total ∆*P*_*C*_ by *L* and [1 − *L*], respectively.) Note that, when 2*α*_f_ *P*_*A*_ is positive, the rate of change of *P*_*C*_ is accelerated. The opposite occurs when 2*α*_f_ *P*_*A*_ is negative.

In the case of an X‐linked locus, gene frequency change equaled that of the autosomal case for females, Δ*P*_*X*,female_ = Δ*P*_*A*,female_ and Δ*P*_*C,*female,X‐linked_ = Δ*P*_*C,*female_. Similarly, selection on the cytoplasmic locus remains the same, but selection in the males on the nuclear locus is weaker.

(3) $$\Delta P_{X,\mathrm{male}}=\frac{1/2(\beta +\alpha_{m}P_{X})}{\left[1+P_{C}\left(\beta +\alpha_{m}P_{X}\right)\right]}P_{X}\left(1-P_{X}\right),$$

(4) $$\Delta P_{C,\mathrm{male},X-\mathrm{linked}}=\frac{\left(\beta +\alpha_{m}P_{X}\right)}{\left[1+P_{C}\left(\beta +\alpha_{m}P_{X}\right)\right]}P_{C}\left(1-P_{C}\right).$$

Unlike the autosomal case where the fitness effects are in range from 1 to 1 + 2*α*, equation (3) has the coefficient 1/2 because the fitnesses of the alternative male genotypes (xY and XY) are 1 and 1 + *α*, respectively.

With a Z‐linked locus, the changes in gene frequency equal those of the autosomal case for the males, Δ*P*_*Z*,male_ = Δ*P*_*A,*male_. However, selection in the females is weaker as was the true for males with X‐linked case, such that Δ*P*_*Z,fem*ale_ = Δ*P*_*X*,male_ and

(5) $$\Delta P_{C,\mathrm{female},Z-\mathrm{linked}}=\frac{\left(\beta +\alpha_{f}P_{Z}\right)}{\left[1+P_{C}\left(\beta +\alpha_{f}P_{A}\right)\right]}P_{C}\left(1-P_{C}\right).$$

We used the measures defined by Asmussen et al. (1987) to investigate how epistatic selection created associations between nuclear and mitochondrial alleles. In particular, we focused on the gametic linkage disequilibrium, which describes the deviation of the frequency of *AC* gametes (*P*_*AC*_) from the expectation under random association (*P*_A_*P_*C*_). This measure, which we will call *LD*, is analogous to often used measures of linkage disequilibrium in multilocus, nuclear models (Hedrick 2005). For the autosomal nuclear case, we derived the expression for the generation of *LD* by selection under the assumption that the initial allele frequencies were independent (*LD* = 0)

(6a) $$\Delta {\mathrm{LD}}_{\mathrm{female}}=\frac{\alpha_{f}}{{\left[1+P_{C}\left(\beta +2\alpha_{f}P_{A}\right)\right]}^{2}}P_{A}\left(1-P_{A}\right)P_{C}\left(1-P_{C}\right),$$

(6b) $$\Delta {\mathrm{LD}}_{\mathrm{male}}=\frac{\alpha_{m}}{{\left[1+P_{C}\left(\beta +2\alpha_{m}P_{A}\right)\right]}^{2}}P_{A}\left(1-P_{A}\right)P_{C}\left(1-P_{C}\right).$$

For the diploid sex, the *LD* generated by selection in the X‐linked and Z‐linked cases (females and males, respectively) is the same as the autosomal case. For the haploid sex, the *LD* created by selection is lower

(7) $$\begin{array}{cc} & \Delta {\mathrm{LD}}_{\mathrm{male},X-\mathrm{linked}}\\ & =1/2\frac{\alpha_{m}}{{\left[1+P_{C}\left(\beta +\alpha_{m}P_{A}\right)\right]}^{2}}P_{X}\left(1-P_{X}\right)P_{C}\left(1-P_{C}\right), \end{array}$$

(8) $$\begin{array}{cc} & \Delta {\mathrm{LD}}_{\mathrm{female},Z-\mathrm{linked}}\\ & =1/2\frac{\alpha_{f}}{{\left[1+P_{C}\left(\beta +\alpha_{f}P_{A}\right)\right]}^{2}}P_{Z}\left(1-P_{Z}\right)P_{C}\left(1-P_{C}\right). \end{array}$$

In equations ((6a), (6b), (7), (8)), the sign of the intergenomic *LD* generated by epistatic selection depends only on the sign of *α*, the selection coefficient of the nuclear allele conditional on cytoplasmic background. Positive *LD* creates a positive association between the mitochondrial allele *C* and the nuclear allele *A*. In the language of indirect genetic effects (Drown and Wade 2014), the nuclear allele, *A*, is favored when in the mitochondrial, heritable environment. And, as *A* increases in frequency, so too does this favorable environment, *C*, owing to positive *LD*. In turn, this increases the strength of selection on *A*, which is represented by (*αP*_*C*_). This, in turn, increases Δ*P*_*C*_ so the coadaptation between *A* and *C* is mutually reinforcing, generating positive feedback or a self‐accelerating runaway process.

### Long‐term dynamics

We explored the evolutionary dynamics of the recursion equations in the main text numerically by forward iteration. For a given set of parameters and initial conditions, we iterated equations ((1a), (1b), (2a), (2b), (3), (4), (5), (6a), (6b), (7), (8)) using MATLAB (R2015b; The Mathworks, Natick, MA, USA) for 5000 generations. We initialized the *A* and *C* alleles at frequency 0.05. With this low initial frequency, we in effect examine how selection would act on a new mutation in the population. At the start of iteration, we assumed that the genotypes were the products of random mating, in multilocus Hardy–Weinberg proportions, and that the sex ratio of the population was maintained at 1:1 males to females. The simulations show the population allele frequency dynamics weighting males and females equally in the autosomal case. For the X‐ and Z‐linked cases, we weighted the haploid sex and diploid sex by 1/3 and 2/3, respectively. We calculated the overall gametic *LD* as the unweighted average of males and females in all cases.

**Figure 1:**
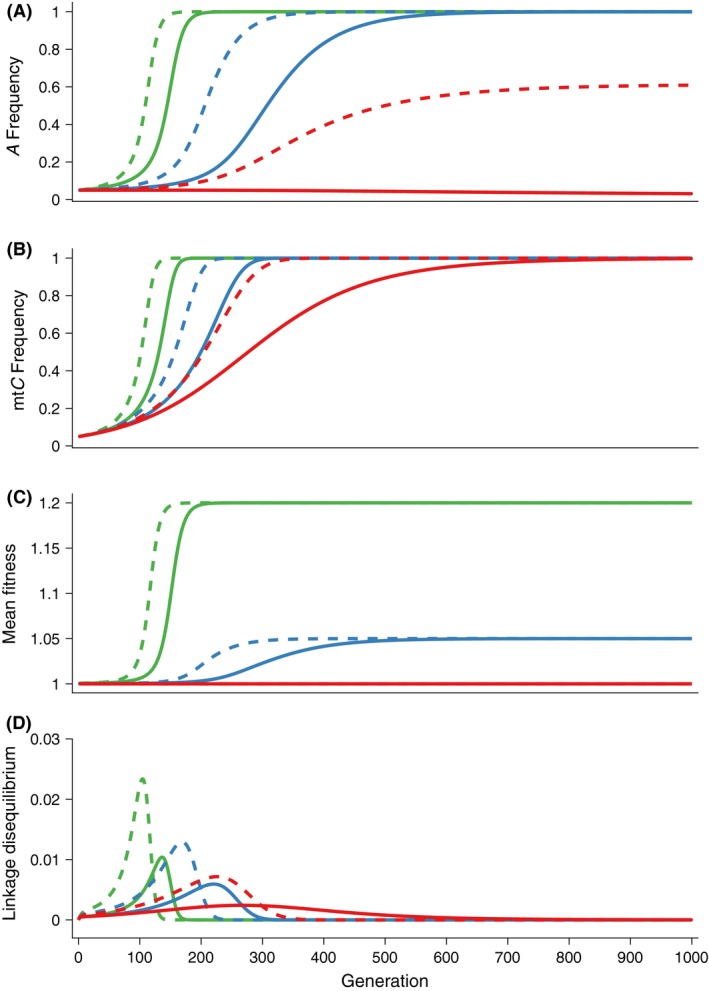
Comparison of the evolutionary dynamics across three cases: genomic cooperation (green lines), genomic cooperation with sexual antagonism (blue lines), and genomic conflict (red lines). For the genomic cooperation case, *α*_f_ = *α*_m_ = 0.1. For the genomic cooperation with sexual antagonism case, *α*_f_ = 0.1 and *α*_m_ = −0.05. For the genomic conflict case, *α*_f_ = 0.1 and *α*_m_ = −0.1. In all cases, the fitness effect of the mitochondrial *C* allele is zero (*β *= 0). (A) Plots of the frequency of the sensitive nuclear allele, *P*_*A*_. (B) Plots of the frequency of the sensitive mitochondrial allele, *P*_*C*_. (C) Plots of the population mean fitness. (D) Plots of the linkage disequilibrium generated between the nuclear and mitochondrial genomes. We contrast simulations without inbreeding (*f *=* *0, solid lines) and with inbreeding (*f *=* *0.5, dashed lines).

We highlight and contrast the possible evolutionary dynamic processes with three examples: (1) genomic cooperation without sexual conflict (0 < *α*_f_ = *α*_m_ = 0.10); (2) genomic cooperation with weak sexual conflict (−0.5*α*_f_ = *α*_m_ < 0 < *α*_f_ = 0.10); and (3) strong genomic and sexual conflict (*α*_f_ = −*α*_m_ < 0 < *α*_f_ = 0.10). Genomic cooperation occurs when both the beneficial nuclear alleles increase in frequency as well as the mitochondrial background upon which they have a benefit. Here, the nuclear alleles increase rapidly in frequency. Via co‐transmission of the mitochondria and the buildup of positive *LD*, the favorable mitochondrial background also increases in frequency in a self‐accelerating or runaway process (Fig. 1, green lines). Notably, for all models, population mean fitness increases monotonically (Fig. 1) as the evolution proceeds. When there is no evolution, mean fitness remains constant (red line).

The introduction of modest sexual conflict (−0.5 *α*_f_ = *α*_m_ < 0 < *α*_f_ = 0.10) slows the runaway process. We see in Figure 1 (blue lines) that again both the nuclear and mitochondrial alleles increase in frequency; however, the rate is reduced compared to the previous case of genomic cooperation without sexual antagonism. Here, the nuclear allele, *A,* on mitochondrial background, *C*, has contrasting fitness effects in females and males: *A* is beneficial for females (*α*_f_ > 0), but weakly deleterious for males (*α*_m_ < 0 and *α*_f_ > −*α*_m_).

When the *A* allele is beneficial for females (*α*_f_ > 0) and equally deleterious for males (*α*_m_ < 0 and *α*_f_ ≤ −*α*_m_), there is a genomic conflict. Because of the beneficial effects of the *C* background on *A* bearing females, the mitochondria‐transmitting sex, the *C* mitochondrial background increases in the population. However, the increase in this background happens in the absence of cooperation from the nuclear genome, and the nuclear allele frequencies do not change (or the *A* allele decreases when *α*_f_ < −*α*_m_). As seen in Figure 1 (red lines), the mitochondrial background, C, increases in frequency, although at a much slower rate than in the other two cases.

We modified our numerical iterations as in Drown and Wade (2014) to allow a fraction, *f*, of within family matings (brother–sister). We revisit the three cases above but with 50% inbreeding (*f* = 0.5) instead of random mating. In both cases of genomic cooperation, the effects of inbreeding accelerate the fixation dynamics. Inbreeding qualitatively changes the evolutionary dynamics of the genomic conflict example (Fig. 1, red‐dashed lines). Inbreeding leads to genomic cooperation in that both the *A* nuclear allele and the *C* mitochondrial allele fix. In general, we find that inbreeding decreases the region of fitness corresponding to genomic conflict.

**Figure 2:**
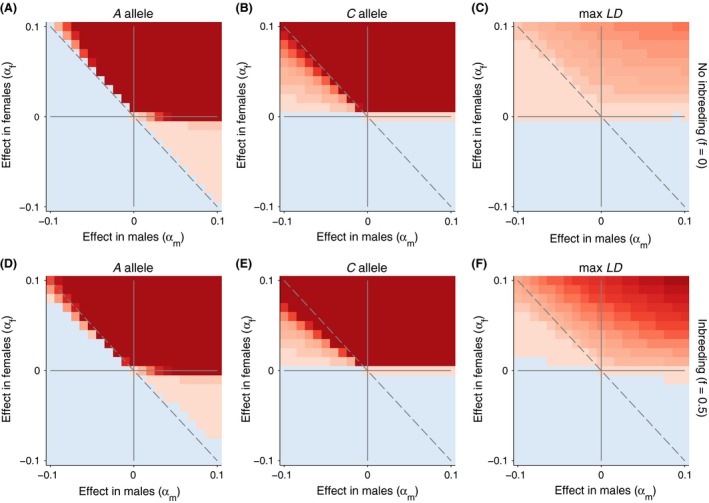
Evolutionary dynamics of the nuclear and mitochondrial loci with an autosomal nuclear allele. In all simulations, *β* is fixed at 0. Allele frequencies that increased after 5000 generations are shown in redder colors. Allele frequencies that decreased are shown in bluer colors. The maximum linkage disequilibrium is plotted on the right with positive values in red and negative values in blue. We contrast cases with and without inbreeding (*f *=* *0, top row) and with inbreeding (*f* = 0.5, bottom row). The magnitude of the color scale is consistent with and without inbreeding.

These three examples provide case studies of the dynamics we see across the entire range of epistatic fitness space. We explored a parameter range of *α* with independent effects for the male and females (*α*_m_ and *α*_f_, respectively) across the range [−0.1, 0.1] in 0.01 increments (Fig. 2). In the first set of simulations, *β* is fixed at 0. After 5000 generations, we calculated the *A* and C allele frequencies. We also calculated the maximum linkage disequilibrium over the course of the 5000 generations of the simulation. We found that, for the majority of cases, the alleles were either fixed for lost from the population. In some cases, we noted that the allele frequencies at one locus remained polymorphic after 5000 generations. However, the terminal allele frequencies were stable across many generations because the other locus, providing the genetic background, had already fixed.

The evolutionary dynamics define four distinct regions in Figure 2 defined by the direction of spread of the nuclear and mitochondrial alleles. We define cooperation and conflict in terms of the population dynamics experienced by each genome. In our model, where individual fitness depends on the interaction between genomes, population mean fitness increases only when *α*_f_ *P*_*A*_ > −*β* and *α*_f_ + *α*_m_ > 0. Here, the changes in frequency of the nuclear allele, *A*, and the mitochondrial allele, *C*, are both positive, and both alleles go to fixation (Fig. 2A and B, upper right quadrant). This occurs even in areas of sexual conflict, where *α*_f_ > 0 but *α*_m_ < 0 (Fig. 2A and B, upper left quadrant).

Sexual conflict characterizes the upper left and lower right quadrants because the gene frequency changes in males and females differ in sign. In a portion of those quadrants, genomic conflict also occurs as either the mitochondrial *C* allele (Fig. 2B, upper left quadrant) or the nuclear *A* allele (Fig. 2A, lower right) slowly increases, while the other is lost. In the case where *α*_m_ > 0, and *α*_m_ > −*α*_f_, then the nuclear *A* allele slowly increases, while *C* is lost (Fig. 2A, lower right quadrant). In the case where *α*_f_ > 0, and *α*_f_ < −*α*_m_, then the mitochondrial *C* allele slowly increases, and *A* is lost (Fig. 2B, upper left quadrant). This region defines an area where, from the gene's eye view of each genome separately, there appears to be genomic cooperation between the mitochondria and the female's nuclear genome. However, this genomic cooperation is outweighed by sexual conflict, resulting in the spread of the mitochondrial background but not the nuclear allele.

The lower left quadrant of Figure 2A and B defines an area of genomic cooperation where gene combination that decreases fitness for both genomes are purged from the population, that is, there is a decrease in both the *A* and *C* allele frequencies. Because the *A* allele decreases fitness on average when on the *C* mitochondrial background, this heritable mitochondrial environment becomes rarer (i.e., *P*_*C*_ decreases) as a result of selection against the *A* allele. That, in turn, weakens future selection on the *A* allele. The net effect is that cooperative genomic selection is weaker at purging deleterious allelic combinations leading to higher frequencies of deleterious genes in both genomes at mutation–selection balance.

#### Intergenomic linkage disequilibrium

The maximum intergenomic *LD* achieved during a simulation for each parameter set is shown in the right most panels of Figure 2, for randomly mating and inbreeding populations, respectively. Inbreeding has two effects on the intergenomic *LD*: (1) It increases the magnitude of positive *LD* above that in a randomly mating population, and (2) it creates positive values of *LD* across a broader range of fitness values than occurring in a randomly mating population. The first effect tends to accelerate intergenomic coevolution, permitting evolutionary change that would not occur with random mating. That is, inbreeding allows some alleles to fix that otherwise would not. An example of this was seen in the trajectory of the *A* allele in the upper panel of Figure 1, where it increases when *f* is 0.50 but not when there is random mating (*f* = 0.0).

#### X‐ and Z‐linked nuclear genes

**Figure 3:**
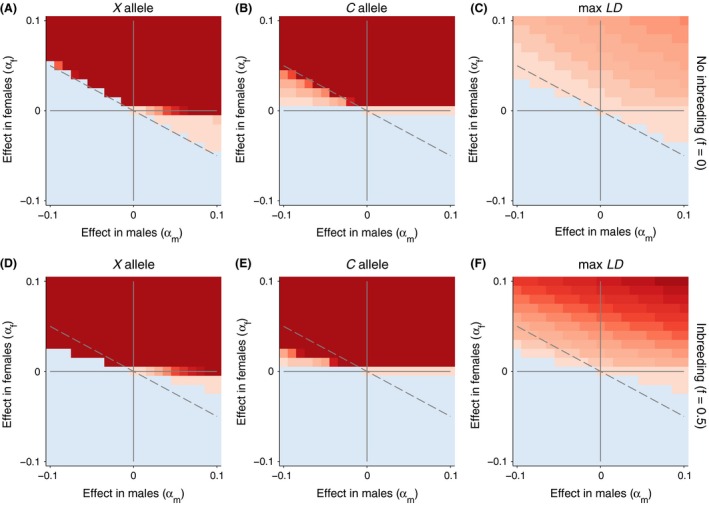
Evolutionary dynamics of the nuclear and mitochondrial loci with an X‐linked nuclear allele. In all simulations, *β* is fixed at 0. Allele frequencies that increased after 5000 generations are shown in redder colors. Allele frequencies that decreased are shown in bluer colors. The maximum linkage disequilibrium is plotted on the right with positive values in red and negative values in blue. We contrast cases with and without inbreeding (*f* = 0, top row) and with inbreeding (*f* = 0.5, bottom row). The magnitude of the color scale is consistent with and without inbreeding.

**Figure 4:**
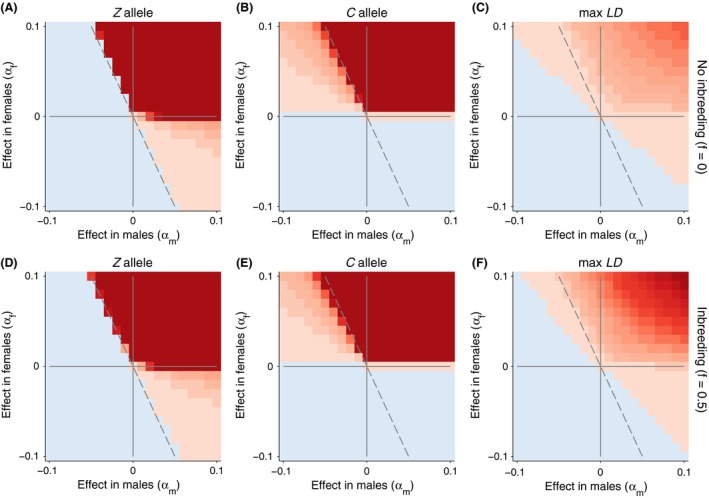
Evolutionary dynamics of the nuclear and mitochondrial loci with a Z‐linked nuclear allele. In all simulations, *β* is fixed at 0. Allele frequencies that increased after 5000 generations are shown in redder colors. Allele frequencies that decreased are shown in bluer colors. The maximum linkage disequilibrium is plotted on the right with positive values in red and negative values in blue. We contrast cases with and without inbreeding (*f* = 0, top row) and with inbreeding (*f* = 0.5, bottom row). The magnitude of the color scale is consistent with and without inbreeding.

To explore the other genomic architectures, we modified our simulation to account for a nuclear allele that was either X‐linked (Fig. 3) or Z‐linked (Fig. 4). In these cases, we used the alternative equations (3), (4), (5) above to calculate allele frequencies. In both cases, the general dynamics are very similar to the autosomal case. The same regions of conflict and cooperation exist, although the areas now differ in size because of the differences in transmission (maternal bias for the X and paternal bias for the Z). For the X‐linked case (Fig. 3), nuclear alleles that are less favorable or unfavorable in males become fixed more often than in the autosomal case because they spend 2/3 of their time in females where they are favored. To emphasize this relationship, we plot a 2/3 ratio line in Figure 3 (dashed gray). A similar shift, but opposite in direction, occurs in the Z‐linked case where Z‐linked alleles spend 2/3 of their time in males. Here, only, weakly deleterious alleles in females relative to males become fixed.

#### Mitochondrial effects on fitness (*β* ≠ 0)

**Figure 5:**
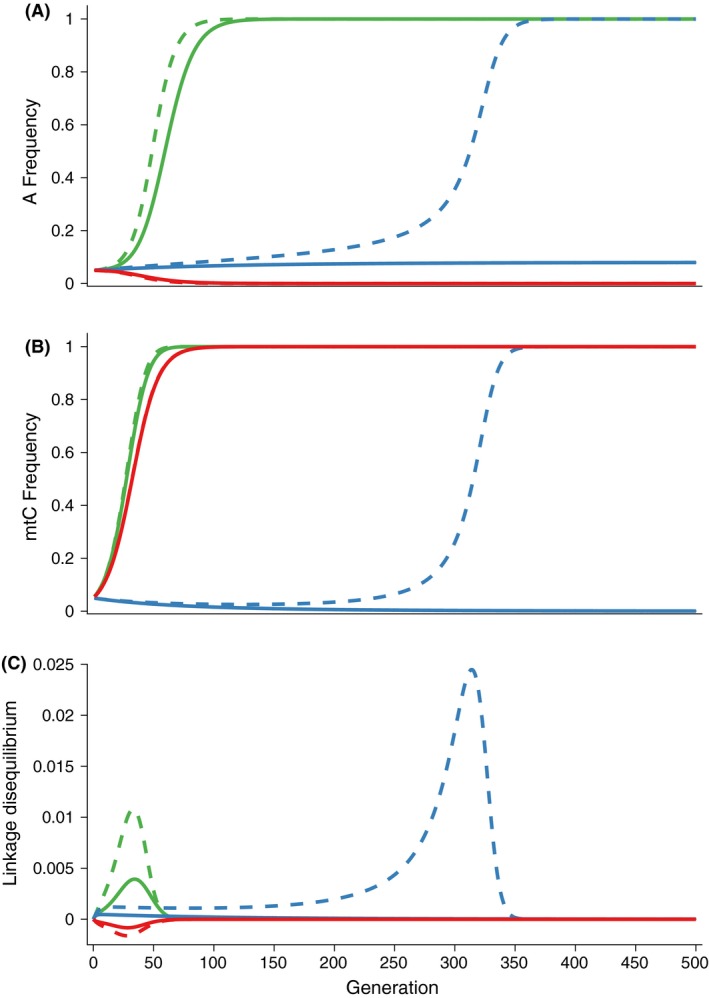
Comparison of the evolutionary dynamics across three cases: genomic cooperation (green lines), genomic cooperation with sexual antagonism (blue lines), and genomic conflict (red lines). For the genomic cooperation case, *α *= *β *= 0.1. For the genomic cooperation with sexual antagonism case, *α *= 0.1 and *β *= −0.025. For the genomic conflict case, *α *= −0.05 and *β *= 0.1. In all cases, the fitness effect of the mitochondrial *C* allele is zero (*β *= 0). (A) Plots of the frequency of the sensitive nuclear allele, *P*_*A*_. (B) Plots of the frequency of the sensitive mitochondrial allele, *P*_*C*_. (C) Plots of the linkage disequilibrium generated between the nuclear and mitochondrial genomes. We contrast simulations without inbreeding (*f *=* *0, solid lines) and with inbreeding (*f *=* *0.5, dashed lines).

We also varied values of *β*, the mitochondrial effect on fitness independent of the nuclear genotype. We highlight and contrast the different dynamic processes with the three examples presented in Figure 5. In the first case (Fig. 5, green lines), the marginal fitnesses of the mitochondrial and nuclear alleles are aligned and positive (*β, α *>* *0). This enhanced degree of genomic cooperation results in the very rapid fixation of both the *A* and *C* alleles: a fixation so rapid that it is only marginally accelerated by inbreeding. In contrast, when we decrease the fitness effect of the autosomal allele by making *α* negative, the *A* allele fails to increase in the population even with the high levels of inbreeding (Fig. 5, red lines). However due to its independent positive effect, the mitochondrial allele goes to fixation at nearly the same rate as the genomic cooperation case (Fig. 5, compare red and green lines).

To illustrate the impact of inbreeding, we present a case just on the cusp of fixation (Fig. 5, blue lines). Here, in a randomly mating population, despite a strongly positive value of *α*, the direct deleterious effect of the mitochondrial background causes it to decrease in the population. As a result, the nuclear allele fails to go to fixation as the deleterious mitochondrial background on which it has a positive fitness effect disappears from the population. However, with inbreeding, the nuclear allele and the mitochondrial background on which it is favorable become linked by family‐level selection. The frequency of the mitochondrial background increases, albeit slowly for the first 150 generations, after which, the large intergenomic *LD* sweeps both the *C* and the *A* alleles to fixation. Here, inbreeding causes the nuclear and mitochondrial gene combination to behave more like a single genetic entity while, with random mating, they evolve almost independently.

**Figure 6:**
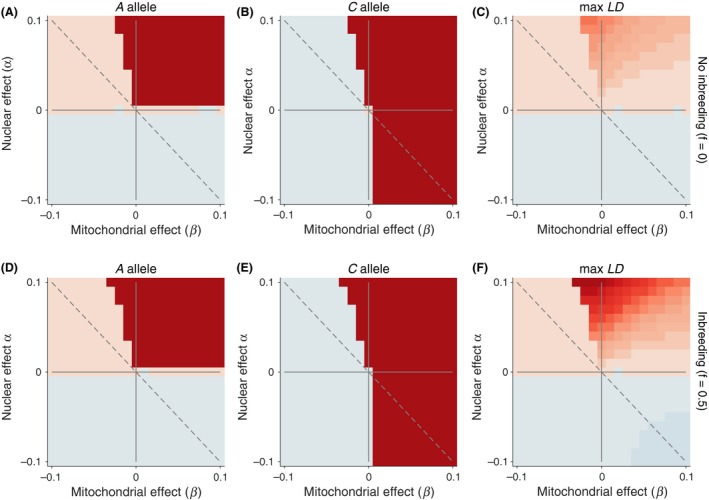
Evolutionary dynamics of the nuclear and mitochondrial loci with an autosomal nuclear allele. In all simulations, *α*_f_ = *α*_m_ = *α*. Allele frequencies that increased after 5000 generations are shown in redder colors. Allele frequencies that decreased are shown in bluer colors. The maximum linkage disequilibrium is plotted on the right with positive values in red and negative values in blue. We contrast cases with and without inbreeding (*f *=* *0, top row) and with inbreeding (*f *=* *0.5, bottom row). The magnitude of the color scale is consistent with and without inbreeding.

In Figure 6, we illustrate that independent, additive fitness effects of the nuclear and mitochondrial alleles predominate over the intergenomic epistatic effects, especially when the former are large. Nevertheless, there are cases where inbreeding is sufficient to maintain the intergenomic *LD* created by epistatic selection. In those cases (dark red region of upper left quadrant), the marginal fitness of a mitochondrial allele with deleterious main effect (i.e., *β *<* *0) can be made positive by its beneficial fitness interaction with the nuclear allele, and the inbreeding‐sustained positive *LD* allows the nuclear–mitochondrial combination to spread to fixation.

## Discussion

With a simple model of nuclear–mitochondrial epistasis, we have two gene's eye views of evolution: one from the perspective of the mitochondrial allele, *C*, and one from the perspective of the nuclear allele, *A*. Whenever the additive effect of the A allele on the C mitochondrial background is deleterious in males (*α*_m_ < 0) and the beneficial in females (0 < *α*_f_), our model results in marginal fitness characteristic of both genomic conflict and sexual conflict. Synergistic fitness effects, both positive and negative, between genomes represent intergenomic epistasis (Dowling et al. 2010; Heath 2010). In our examples, the intergenomic epistasis occurs between genes in the nucleus and the mitochondria, extending the models of classical, maternal–zygotic, and sib–sib epistasis of Drown and Wade (2014). As in those models, a single genetic background, the mitochondrial, generated a fitness advantage or disadvantage for one of two nuclear alleles. Positive intergenomic epistasis for fitness created intergenomic *LD*, a nonrandom association between the two genomes within a single population. The mating system, random mating or inbreeding via brother–sister mating, significantly influences whether the *LD* created by epistatic selection is sustained. Importantly, there are cases where the presence of inbreeding allows epistasis to change the marginal fitness of either the nuclear or the mitochondrial allele from negative to positive, resulting in the fixation of alleles that would otherwise be lost with random mating. The rate of change of mean fitness is always positive and monotonic when there is an evolutionary change in gene frequency. However, mean fitness increases rapidly under cooperation where there are positive, synergistic fitness effects but much more slowly where conflict is predominant.

In contrast, under the gene's eye view of evolution, evolution in one genome tends to be perceived as independent of the dynamics in the other, as though intergenomic conflict had no synergistic component. Moreover, with models that assume independent mitochondrial fitnesses, mating system (here inbreeding or outbreeding) has no effect on the evolutionary dynamic. Advocates of the gene's eye view (Williams 1966; Dawkins 1976) have argued that, once an allele's marginal fitness has been calculated, evolution can be treated as though interactions with other genes, genomes, or the environment are of no further consequence (see, e.g., Williams 1966, p. 56). Without the comprehensive multiple gene perspective, no evolutionary change can predict the interaction we have modeled. Specifically, when there is epistasis for fitness, selection creates nonrandom associations between alleles, in this case between alleles in different genomes. These transgenomic associations accelerate the evolutionary dynamics when positive and decelerate them when negative. As a result, theoretical or empirical focus on a single gene in either genome, as advocated by the gene's eye view, misses an important aspect of the coevolutionary dynamics of both genomes when there is epistasis for fitness. In the absence of epistasis for fitness, the gene's eye view may suffice. Our analytical solutions as well as the numerical simulations demonstrate that, owing to synergistic nuclear–mitochondrial epistasis, positive intergenomic linkage disequilibrium is generated. This *LD* accelerates the evolutionary change in allele frequencies beyond what might be predicted examining the two genes independently. We believe that a focus on genic independence has led to an emphasis on genomic conflict (e.g., Rice 2013) at the expense of consideration of genomic cooperation, about which we know relatively little.

The evolutionary dynamics of intergenomic cooperation appear to be very different from those of intergenomic conflict. Positive transgenomic fitness effects lead to rapid and self‐accelerating fixation of alleles in both genomes. In contrast, negative transgenomic fitness effects lead to extremely slow and decelerating evolutionary dynamics where neither allele fixes. We can apply Falconer's (1981) argument for a negative genetic correlation between fitness components, such as viability and fecundity, to genomic interactions which affect fitness of alleles in both genomes. Falconer argued that pleiotropic genes with positive fitness effects on both viability and fecundity experience strong directional selection and fix rapidly. Conversely, those pleiotropic genes with negative effects on both fitness components experience strong purifying selection and are lost from the population. However, those genes with positive effects on one fitness component and negative effects on the other experience much weaker total selection and thus remain at intermediate frequencies in a population for a longer period of time. He argued that was these genes with antagonistic pleiotropy that remain segregating in the population that result in a negative genetic covariance between components of fitness (Falconer 1981, p. 300). A similar argument can be applied to epistatic fitness interactions (Wade 1992), such as those between the nuclear and mitochondrial genomes. If alleles at a nuclear and a mitochondrial locus each contributes positively to fitness and their interaction is also positive, then these alleles will experience strong and accelerating selection toward fixation (see upper right quadrant of Fig. 6). Conversely, if both have negative main effects on fitness and negative interactions with respect to fitness, both alleles will rapidly be lost from the population (see lower left quadrant of Fig. 6). Genes whose main effects and interactions are of opposite sign will be maintained in the population for a longer period of time. Hence, the standing genetic variance will contain low‐frequency alleles with conflicting fitness effects across genomes. However, the standing variance is not representative of the adaptive process which depends upon those rarer synergistic variants that are swept rapidly through (or out of) populations.

Niche construction theory (Odling‐Smee et al. 2003) and the theory of indirect genetic effects (Wolf et al. 1998) provide a more cogent framework for interpreting our results. Both emphasize dual inheritance and how change in one entity, genetic or environmental, can alter the adaptive environment of another. In our model, as the *A* allele increases in frequency, it increases the frequency of the mitochondrial environment (i.e., *C*) on which it enjoys a fitness advantage (Figs. 1, 5). Because inbreeding accelerates the increase in *A* allele frequency, it also increases the rate of change of the mitochondrial environment on which it is adaptive. Inbreeding can be interpreted as increasing the fidelity of the co‐inheritance of the *A* allele and the mitochondrial background on which it is adaptive. The increase in intergenomic *LD* with inbreeding is evidence of this association.

While adaptation can occur by either conflict or cooperation, we have shown that the evolutionary dynamics of adaptation via cooperation occur much more rapidly than those with conflict. The adaptive change with genomic cooperation is a runaway process, similar to Fisherian sexual selection via female mate choice (Fisher 1958; Lande 1981; Kirkpatrick 1982; Drown and Wade 2014). Here, an increase in allele frequency in one genome increases the strength of selection in the other. The evolutionary dynamics of conflict are fundamentally different, because successful alleles in one genome lower the frequency of the genetic background in the other genome on which their selective success depends. Differently put, with conflict, the genomic environment in one genome favoring an allele in the other becomes rare in inverse proportion to the frequency of that allele. Although the gene's eye view of evolution perceives conflict as the inevitable outcome of separate genetic entities pursuing independent evolutionary interests, it is not what happens here. Differently put, the ability to calculate a marginal mean fitness for an allele embedded in a complex network of interaction does not license treating that gene as an independent entity.

The genetic architecture of genomic interactions alters the landscape of adaptation. We found that for autosomal nuclear genes, the regions of genomic conflict are smaller than those of genomic cooperation. Moving the nuclear gene to an X‐linked region reduces the region of genomic conflict. Conversely, the region of conflict is enlarged for Z‐linked nuclear genes. We note that Z‐linked nuclear genes interacting with mitochondrial genes could underlie the energetic courtship behaviors which characterize many bird taxa. Conversely, the genetic architecture of X‐linked traits and haplo‐diploid species mitigates nuclear–mitochondrial conflict.

Our analyses identified the generation of positive *LD* between genomes as contributing to a runaway process of cooperative coevolution. In some two‐locus models and as seen here, synergistic selection creates positive *LD* from an initial random association where *LD* is zero (Felsenstein 1981). Epistatic selection and recombination act as opposing processes, creating and destroying *LD*, respectively (Charlesworth and Charlesworth 2010, p. 425). In an earlier study, we illustrated that the role of inbreeding and population structure is important to runaway adaptive processes (Drown and Wade 2014). Our numerical results show that increased inbreeding not only increases the rates of nuclear–mitochondrial coadaptation, but also increases the size of the region of genomic cooperation.

We can draw an analogy between our results for genomic conflict and ecological competition theory, particularly competitive exclusion. Consider two species that occupy the same ecological niche and are in competition (i.e., conflict) for a shared resource. When considered separately (the gene's eye view), they either would occupy the niche in the absence of the other competitor, but when both species are present, only the most competitive wins, while the inferior competitor is excluded. This scenario parallels the findings of our genetic model. Here, the shared resource or niche is organismal fitness; both genomes evolve by enhancing the fitness of single individuals. With genomic conflict, like competition between species, the success of one genome occurs at the expense of the other. In the regions of genomic conflict identified in Figures 1 and 2, the allele in one genome spreads, while the interacting allele in the other genome is lost. With mutation, a “lost allele” in either genome can be maintained at a low frequency, just like an excluded species can be maintained by immigration. This adaptive “genetic exclusion” as the outcome of genomic conflict is analogous to competitive exclusion as a result of ecological resource conflict.

## Conclusion

The process of coevolution between mitochondrial and nuclear genomes has also been viewed as a neutral process from the perspective of the mitochondrion. Its haploid asexual life history and greatly reduced effective population size (Blanchard and Lynch 2000) reduce the efficacy of selection and eventually result in the loss of gene function, genome reduction, and the transfer of mitochondrial genes to the nucleus. This process has been called “a key step in stabilizing the transition from an autonomous endosymbiont to a host‐dependent mitochondrion” (Lynch 2007, p. 308). However, Brandvain and Wade (2009) showed that frequent mitochondrial to nuclear gene transfer requires synergistic epistatic selection and occurs only rarely when driven by mutation and drift.

We suggest that, although genomic conflict is possible, these theoretical restrictions limit the ability of the conflict paradigm to account for the major features of mitochondrial and nuclear coevolution. Similar synergistic fitness interactions have recently been shown to be essential for the evolution of vertical from horizontal transmission (Drown et al. 2013).

## Conflict of Interest

The authors declare that there are no conflict of interests.
